# Supplementary material for: Association between glucose-to-lymphocyte ratio and in-hospital mortality in acute myocardial infarction patients
Source: PLoS One. 2023 Dec 7;18(12):e0295602. doi: 10.1371/journal.pone.0295602 (PMC10703328; doi:10.1371/journal.pone.0295602)
Supplement: S1 File — (DOCX) [file pone.0295602.s001.docx]

**Table S1. Description of missing variables**

| Variables | Numbers | Proportion % |
| --- | --- | --- |
| GCS | 1 | 0.06 % |
| RDW | 1 | 0.06 % |
| Neutrophil | 1 | 0.06 % |
| Platelet | 2 | 0.11 % |
| SBP | 14 | 0.78 % |
| HR | 17 | 0.94 % |
| MBP | 25 | 1.39 % |
| DBP | 26 | 1.44 % |
| SPO_2_ | 40 | 2.22 % |
| PTT | 41 | 2.27 % |
| PT | 44 | 2.44 % |
| INR | 44 | 2.44 % |
| Temperature | 146 | 8.09 % |
| RR | 176 | 9.75 % |
| Weight | 187 | 10.36 % |
| Lactate | 311 | 17.23 % |

GCS: Glasgow Coma Scale score, RDW: red cell distribution width, SBP: systolic blood pressure, HR: heart rate, MBP: mean blood pressure, DBP: diastolic blood pressure, PTT: partial thromboplastin time, PT: prothrombin time, INR: international normalized ratio, RR: respiratory rate.

**Table S2. Sensitivity analysis of characteristics of patients before and after interpolation of missing variables**

| Variables | After interpolation (n=1805) | Before interpolation (n=1805) | Statistics | *P* |
| --- | --- | --- | --- | --- |
| GCS score, Mean ± SD | 12.35 ± 3.91 | 12.35 ± 3.91 | t=0.00 | 0.999 |
| RDW, %, Mean ± SD | 14.52 ± 1.98 | 14.52 ± 1.98 | t=0.04 | 0.970 |
| Neutrophil, %, Mean ± SD | 79.26 ± 11.22 | 79.28 ± 11.19 | t=-0.06 | 0.955 |
| Platelet, K/uL, M (Q_1_, Q_3_) | 177.00 (130.00, 236.00) | 177.00 (130.00, 236.00) | Z=-0.022 | 0.982 |
| SBP, mmHg, Mean ± SD | 118.71 ± 21.77 | 118.63 ± 21.78 | t=0.12 | 0.907 |
| HR, bpm, Mean ± SD | 85.67 ± 16.02 | 85.60 ± 16.06 | t=0.13 | 0.895 |
| MBP, mmHg, Mean ± SD | 80.84 ± 16.32 | 80.60 ± 16.15 | t=0.43 | 0.664 |
| DBP, mmHg, Mean ± SD | 64.04 ± 15.94 | 63.78 ± 15.74 | t=0.48 | 0.630 |
| SPO_2_, %, Mean ± SD | 97.55 ± 3.02 | 97.57 ± 3.05 | t=-0.25 | 0.802 |
| PTT, seconds, M (Q_1_, Q_3_) | 33.20 (28.20, 47.10) | 33.00 (28.10, 46.45) | Z=-0.553 | 0.580 |
| PT, seconds, M (Q_1_, Q_3_) | 14.70 (13.10, 16.90) | 14.80 (13.10, 17.00) | Z=0.199 | 0.842 |
| INR, M (Q_1_, Q_3_) | 1.37 (1.20, 1.50) | 1.30 (1.20, 1.60) | Z=-0.003 | 0.997 |
| Temperature, ℃, Mean ± SD | 36.56 ± 0.64 | 36.56 ± 0.66 | t=-0.27 | 0.785 |
| RR, bpm, Mean ± SD | 18.24 ± 5.21 | 18.37 ± 5.41 | t=-0.73 | 0.467 |
| Weight, kg, Mean ± SD | 83.10 ± 18.42 | 83.08 ± 19.11 | t=0.03 | 0.979 |
| Lactate, mmol/L, M (Q_1_, Q_3_) | 1.90 (1.41, 2.60) | 1.90 (1.40, 2.80) | Z=0.534 | 0.593 |

GCS: Glasgow Coma Scale score, SD: standard deviation, RDW: red cell distribution width, M: median, Q_1_:1st quartile, Q_3_:3rd quartile, SBP: systolic blood pressure, HR: heart rate, MBP: mean blood pressure, DBP: diastolic blood pressure, PTT: partial thromboplastin time, PT: prothrombin time, INR: international normalized ratio, RR: respiratory rate.

t: t test, Z: rank sum test.
