# Supplementary material for: Association between glucose-to-lymphocyte ratio and in-hospital mortality in acute myocardial infarction patients
Source: PLoS One. 2023 Dec 7;18(12):e0295602. doi: 10.1371/journal.pone.0295602 (PMC10703328; doi:10.1371/journal.pone.0295602)
Supplement: Attachment — Submitted filename: Manuscript.docx [file pone.0295602.s002.docx]

**Association between glucose-to-lymphocyte ratio and in-hospital mortality in acute myocardial infarction patients**

Jing Liu^1*^, Xiaogang Hu^2^

^1^ Department of Emergency (West Branch), The Second Hospital of Shanxi Medical University, Taiyuan 030053, Shanxi, P.R. China

^2^ Department of Internal Medicine, Shanxi Agricural University Hospital, Taiyuan 030031, Shanxi, P.R. China

***Corresponding author:** Jing Liu, Department of Emergency (West Branch), The Second Hospital of Shanxi Medical University, No.379 Yingze West Street, Wanbailin District, Taiyuan 030053, Shanxi, P.R. China

Tel: +86-13753150211

E-mail: liujingEmer@outlook.com

**Abstract**

**Background:** Inflammation is involved in the development and progression of atherosclerosis. Recent studies indicated that of glucose-to-lymphocyte ratio (GLR) level were significantly associated with an increased risk of mortality from inflammatory diseases and showed a specific prognostic value. Herein, this study intended to analyze the association between GLR level and the risk of in-hospital mortality in acute myocardial infarction (AMI) patients and evaluate the predictive value of GLR on the prognosis of AMI.

**Methods:** This retrospective cohort study collected data from 1,805 adult AMI patients with information on glucose and lymphocytes in the Medical Information Mart for Intensive Care IV (MIMIC-IV) database from 2012 to 2019. The associations between GLR and in-hospital mortality were evaluated using univariate and multivariate COX proportional hazard model analyses, with hazard ratios (HRs) and 95% confidence intervals (CIs). Subgroups based on vasopressor use, SOFA scores, renal replacement therapy, gender, coronary artery bypass graft, β blockers and age were used for further exploration. We also compared the effect of GLR, glucose and lymphocytes on predicting in-hospital mortality in AMI patients.

**Results:** Of the total study population, 248 (13.74%) patients died in the hospital. The results showed that high GLR level was associated with an increased risk of in-hospital mortality in AMI patients [HR=1.70, 95% CI: (1.24-2.34)]. In patients who were male, aged ≥ 65 years old, did not have renal replacement therapy, coronary artery bypass graft, or β blockers, had different SOFA scores, and used/not use vasopressor, this relationship was also found (all *P*<0.05). Moreover, the predictive value of GLR in in-hospital mortality was superior to glucose or lymphocytes alone.

**Conclusion:** GLR may be a potential predictor for AMI, which can help identify and manage high-risk populations early in clinical.

**Keywords:** AMI, GLR, MIMIC-IV, retrospective cohort study

**Introduction**

Acute myocardial infarction (AMI) is myocardial necrosis most often caused by persistent ischemia and hypoxia resulting from rupture or erosion of atherosclerotic plaques [1]. AMI is a common disease in intensive care units (ICUs) and a significant cause of acute mortality worldwide [2]. Although the application of interventional therapy and the improvement of drugs have partly improved the prognosis of AMI patients, the number of hospitalized AMI patients has increased in the past decade in China. At the same time, the in-hospital mortality has not decreased in the same period [3, 4]. It is critical to measure biomarker concentrations soon after the onset of AMI, which might contribute to the early identification of patients at high risk of poor results and help further clinical treatment [5]. Regrettably, current prognostic indicators for AMI, such as C-reactive protein and brain natriuretic peptide, are slow and costly [6]. Therefore, uncovering novel predictors that are faster and easier to access is imperative.

Inflammation is involved in the development and progression of atherosclerosis [7]. Lymphocyte count plays a crucial role in regulating inflammatory responses at all stages of atherosclerosis [8] and is associated with AMI occurrence and prognosis [9]. In the early stage of AMI, hyperglycemia promotes prethrombotic status, increases inflammation and sympathetic nervous system activity, worsens endothelial function, induces oxidative stress to release reactive oxygen species, and thus exacerbates coronary artery damage [10, 11]. Paolisso et al. [12] showed that hyperglycemia is associated with higher mortality in AMI patients, independent of diabetes status. Glucose-to-lymphocyte ratio (GLR) is a new marker of inflammation load. Studies indicated that high levels of GLR were significantly associated with an increased risk of mortality from inflammatory diseases such as cancer and acute respiratory distress syndrome (ARDS) and showed a specific prognostic value [13-15]. However, no studies have investigated the association between GLR and mortality risk in AMI patients.

Herein, this study intended to analyze the association between GLR level and the risk of in-hospital mortality in AMI patients based on the Medical Information Mart for Intensive Care IV (MIMIC-IV) database and evaluated the predictive value of GLR on the prognosis of AMI to screen out a powerful marker to assist AMI risk stratification.

**Methods**

**Study design and population**

Data in this retrospective cohort study were extracted from the MIMIC-IV database, which builds upon the success of MIMIC-III and incorporates numerous improvements over MIMIC-III (https://mimic.mit.edu/docs/iv/). MIMIC-IV contains actual hospital stays for patients admitted to a tertiary academic medical center in Boston, MA, USA, between 2012 and 2019 and is intended to support a wide variety of research in healthcare. Comprehensive information for each patient while they were in the hospital, such as laboratory measurements, medications administered, and vital signs were documented and included in MIMIC-IV [16]. This database is approved by Massachusetts Institute of Technology and the Institutional Review Board of Beth Israel Deaconess Medical Center (BIDMC) [17]. The requirement of ethical approval for this was waived by the Institutional Review Board of The Second Hospital of Shanxi Medical University because the data was accessed from the MIMIC-IV database (a publicly available database). The Institutional Review Board of The Second Hospital of Shanxi Medical University waived the need for written informed consent due to the study's retrospective nature.

This study involved adult AMI patients who were diagnosed with an International Classification of Diseases, 9th revision (ICD-9) code of 41000-41092 or an International Classification of Diseases, 10th revision (ICD-10) code of I210.0-I214.0 or I219.0 (the ICD code of AMI) [6]. The exclusion criteria were as follows: (1) age <18 years old, (2) missing information on glucose and lymphocyte count, and (3) hospitalized in the ICU for less than 24 hours.

**Variable collection**

The variables included (1) demographic characteristics such as age, gender, race, insurance and weight, (2) treatment including ventilation use, vasopressor use, renal replacement therapy (RRT), percutaneous coronary intervention (PCI), coronary artery bypass graft (CABG) and β blockers use, (3) comorbidities such as Charlson Comorbidity Index (CCI), congestive heart failure, atrial fibrillation, cardiogenic shock, diabetes and hypertension (identified using ICD-9 codes), (4) vital signs including systolic blood pressure (SBP), diastolic blood pressure (DBP), mean blood pressure (MBP), respiratory rate, heart rate, temperature and SPO_2_, (5) scoring systems like Simplified Acute Physiology Score II (SAPS-II), Sequential Organ Failure Assessment (SOFA) score and Glasgow Coma Scale (GCS) score, (6) laboratory inspection including white blood cell (WBC), neutrophil, platelet, hemoglobin, red cell distribution width (RDW), creatinine, international normalized ratio (INR), prothrombin time (PT), partial thromboplastin time (PTT), blood urea nitrogen (BUN), lactate, bicarbonate, sodium, potassium and chloride. Moreover, the GLR was calculated as follows: GLR = glucose (mg/dL) ÷ lymphocytes count (K/uL). In this study, the median GLR was set as the truncation value (106.14), GLR level >106.14 was defined as the high group, and the GLR <106.14 was defined as the low group. Information was recorded at the admission of the ICU, and only data from the first ICU after the first admission was collected for multiple hospitalizations.

**Outcomes and follow-up**

The outcomes were in-hospital mortality of AMI patients in our study. In-hospital information was recorded by the hospital department in the MIMIC-Ⅳ database, and Social Security Bureau recorded out-of-hospital information. Therefore, the death information of the patients is recorded in self-case. The follow-up endpoint is the end of 2020. The start time of follow-up was the first time of ICU admission, and the end time of follow-up was the time of discharge or death in the hospital.

**Statistical analysis**

Normally distributed measurement data were described by mean ± standard deviation (Mean ± SD) and using a t-test to compare the difference between the two groups. Skewed distributed data were described by median and quartile [M (Q1, Q3)], and the Wilcoxon rank sum test was used to compare the difference between the two groups. The composition ratio [N (%)] was used to describe the distribution of categorical data, and the chi-square test or Fisher’s exact test was used to compare the differences between groups. Univariate and multivariate COX proportional hazard model was established to explore the association between GLR and in-hospital mortality in AMI patients. We used the restricted cubic spline (RCS) curve to describe the relationship between GLR and the risk of in-hospital mortality occurrence. Kaplan-Meier (KM) curve was used to analyze the association between GLR and survival probability in AMI patients. The evaluation indexes were hazard ratios (HRs) with confidence intervals (CIs). *P*<0.05 indicates a significant difference. Missing variables were deleted if the proportion of missing value > 20%; otherwise, they were interpolated using the random forest interpolation method (Table S1). Sensitivity analysis on the data before and after interpolation is shown in Table S2. Statistics analysis was completed by SAS 9.4 (SAS Institute., Cary, NC, USA) and R version 4.2.1 (2022-06-23 ucrt).

**Results**

**Characteristics of AMI patients**

Figure 1 was the flowchart of the data screening. A total of 2022 adult AMI patients with the information of glucose and lymphocytes were absorbed in this study. After excluding 197 patients were hospitalized in the ICU for less than 24 hours, there were 1805 eligible patients enrolled in the analysis. The comparison of the characteristics of AMI patients with different levels of GLR is shown in Table 1. Finally, 248 (13.74%) patients died in the hospital. The median follow-up time was 6.24 (4.33, 10.81) days. The average age of the total population was 68.78±12.57 years old, and 1196 (66.28%) were males. There were 173 (9.58%), 185 (10.25%) and 239 (13.24%) patients who received renal replacement therapy, percutaneous coronary intervention and coronary artery bypass graft treatment, respectively. The median glucose, lymphocytes and GLR in the low GLR group and high GLR group were 127 vs. 171 (mg/dL), 2.03 vs. 0.85 (K/uL) and 66.17 vs. 198.41, respectively.

**Table 1. Comparison of the characteristics of AMI patients with different levels of GLR**

| Variables | Total  (n=1805) | Low GLR level  (n=902) | High GLR level  (n=903) | Statistics | *P* |
| --- | --- | --- | --- | --- | --- |
| Age, years, Mean ± SD | 68.78 ± 12.57 | 67.26 ± 12.46 | 70.30 ± 12.51 | t=-5.17 | <0.001 |
| Gender, n (%) |  |  |  | χ^2^=0.186 | 0.666 |
| Female | 609 (33.74) | 300 (33.26) | 309 (34.22) |  |  |
| Male | 1196 (66.26) | 602 (66.74) | 594 (65.78) |  |  |
| Race, n (%) |  |  |  | χ^2^=2.947 | 0.400 |
| White | 1165 (64.54) | 565 (62.64) | 600 (66.45) |  |  |
| Black | 102 (5.65) | 54 (5.99) | 48 (5.32) |  |  |
| Others | 170 (9.42) | 91 (10.09) | 79 (8.75) |  |  |
| Unknown | 368 (20.39) | 192 (21.29) | 176 (19.49) |  |  |
| Insurance, n (%) |  |  |  | χ^2^=10.123 | 0.006 |
| Medicaid | 77 (4.27) | 42 (4.66) | 35 (3.88) |  |  |
| Medicare | 940 (52.08) | 436 (48.34) | 504 (55.81) |  |  |
| Others | 788 (43.66) | 424 (47.01) | 364 (40.31) |  |  |
| Weight, kg, Mean ± SD | 83.10 ± 18.42 | 84.31 ± 18.40 | 81.89 ± 18.37 | t=2.80 | 0.005 |
| Ventilation, n (%) |  |  |  | χ^2^=0.039 | 0.844 |
| No | 118 (6.54) | 60 (6.65) | 58 (6.42) |  |  |
| Yes | 1687 (93.46) | 842 (93.35) | 845 (93.58) |  |  |
| Vasopressor, n (%) |  |  |  | χ^2^=0.577 | 0.448 |
| No | 678 (37.56) | 331 (36.70) | 347 (38.43) |  |  |
| Yes | 1127 (62.44) | 571 (63.30) | 556 (61.57) |  |  |
| Renal replacement therapy, n (%) |  |  |  | χ^2^=37.810 | <0.001 |
| No | 1632 (90.42) | 854 (94.68) | 778 (86.16) |  |  |
| Yes | 173 (9.58) | 48 (5.32) | 125 (13.84) |  |  |
| Percutaneous coronary intervention, n (%) |  |  |  | χ^2^=22.335 | <0.001 |
| No | 1620 (89.75) | 840 (93.13) | 780 (86.38) |  |  |
| Yes | 185 (10.25) | 62 (6.87) | 123 (13.62) |  |  |
| Coronary artery bypass graft, n (%) |  |  |  | χ^2^=53.304 | <0.001 |
| No | 1566 (86.76) | 730 (80.93) | 836 (92.58) |  |  |
| Yes | 239 (13.24) | 172 (19.07) | 67 (7.42) |  |  |
| Beta blockers, n (%) |  |  |  | χ^2^=0.386 | 0.534 |
| No | 1317 (72.96) | 664 (73.61) | 653 (72.31) |  |  |
| Yes | 488 (27.04) | 238 (26.39) | 250 (27.69) |  |  |
| Charlson comorbidity index, score, M (Q_1_, Q_3_) | 3.00 (2.00, 5.00) | 3.00 (2.00, 4.00) | 4.00 (2.00, 6.00) | Z=-9.541 | <0.001 |
| Congestive heart failure, n (%) |  |  |  | χ^2^=52.936 | <0.001 |
| No | 924 (51.19) | 539 (59.76) | 385 (42.64) |  |  |
| Yes | 881 (48.81) | 363 (40.24) | 518 (57.36) |  |  |
| Atrial fibrillation, n (%) |  |  |  | χ^2^=1.160 | 0.281 |
| No | 1286 (71.25) | 653 (72.39) | 633 (70.10) |  |  |
| Yes | 519 (28.75) | 249 (27.61) | 270 (29.90) |  |  |
| Cardiogenic shock, n (%) |  |  |  | χ^2^=52.271 | <0.001 |
| No | 1545 (85.60) | 826 (91.57) | 719 (79.62) |  |  |
| Yes | 260 (14.40) | 76 (8.43) | 184 (20.38) |  |  |
| Diabetes, n (%) |  |  |  | χ^2^=11.161 | <0.001 |
| No | 1079 (59.78) | 574 (63.64) | 505 (55.92) |  |  |
| Yes | 726 (40.22) | 328 (36.36) | 398 (44.08) |  |  |
| Hypertension, n (%) |  |  |  | χ^2^=0.141 | 0.708 |
| No | 648 (35.90) | 320 (35.48) | 328 (36.32) |  |  |
| Yes | 1157 (64.10) | 582 (64.52) | 575 (63.68) |  |  |
| Systolic, mmHg, Mean ± SD | 118.71 ± 21.77 | 116.94 ± 19.73 | 120.49 ± 23.51 | t=-3.48 | <0.001 |
| Diastolic, mmHg, Mean ± SD | 64.04 ± 15.94 | 62.67 ± 14.55 | 65.41 ± 17.11 | t=-3.66 | <0.001 |
| MBP, mmHg, Mean ± SD | 80.84 ± 16.32 | 80.04 ± 14.77 | 81.63 ± 17.71 | t=-2.08 | 0.038 |
| Respiratory rate, bpm, Mean ± SD | 18.24 ± 5.21 | 17.06 ± 4.63 | 19.41 ± 5.49 | t=-9.82 | <0.001 |
| Heart rate, bpm, Mean ± SD | 85.67 ± 16.02 | 83.52 ± 14.29 | 87.81 ± 17.33 | t=-5.74 | <0.001 |
| Temperature, ℃, Mean ± SD | 36.56 ± 0.64 | 36.50 ± 0.58 | 36.61 ± 0.69 | t=-3.60 | <0.001 |
| SPO_2_, %, Mean ± SD | 97.55 ± 3.02 | 98.06 ± 2.71 | 97.03 ± 3.23 | t=7.37 | <0.001 |
| SAPS-II scores, M (Q_1_, Q_3_) | 38.00 (30.00, 48.00) | 36.00 (29.00, 44.00) | 41.00 (32.00, 52.00) | Z=-8.323 | <0.001 |
| SOFA score, M (Q_1_, Q_3_) | 6.00 (4.00, 9.00) | 5.00 (3.00, 8.00) | 7.00 (4.00, 10.00) | Z=-7.668 | <0.001 |
| GCS score, M (Q_1_, Q_3_) | 14.00 (12.00, 15.00) | 14.00 (13.00, 15.00) | 14.00 (10.00, 15.00) | Z=7.172 | <0.001 |
| WBC, K/uL, M (Q_1_, Q_3_) | 12.80 (9.50, 17.10) | 13.30 (10.10, 17.20) | 12.31 (8.60, 17.00) | Z=3.894 | <0.001 |
| Neutrophil, %, Mean ± SD | 79.26 ± 11.22 | 74.64 ± 10.87 | 83.88 ± 9.54 | t=-19.20 | <0.001 |
| Platelet, K/uL, M (Q_1_, Q_3_) | 177.00 (130.00, 236.00) | 169.50 (128.00, 223.00) | 186.00 (131.00, 250.00) | Z=-3.467 | <0.001 |
| Hemoglobin, g/Dl, Mean ± SD | 10.71 ± 2.50 | 10.68 ± 2.53 | 10.74 ± 2.48 | t=-0.54 | 0.587 |
| RDW, %, Mean ± SD | 14.52 ± 1.98 | 14.26 ± 1.93 | 14.78 ± 2.00 | t=-5.57 | <0.001 |
| Creatinine, mg/dL, M (Q_1_, Q_3_) | 1.10 (0.80, 1.70) | 1.00 (0.80, 1.30) | 1.30 (0.90, 2.10) | Z=-11.118 | <0.001 |
| INR, M (Q_1_, Q_3_) | 1.37 (1.20, 1.50) | 1.40 (1.20, 1.50) | 1.30 (1.20, 1.50) | Z=3.173 | 0.002 |
| PT, seconds, M (Q_1_, Q_3_) | 14.70 (13.10, 16.90) | 15.10 (13.40, 16.90) | 14.38 (12.90, 16.90) | Z=3.372 | <0.001 |
| PTT, seconds, M (Q_1_, Q_3_) | 33.20 (28.20, 47.10) | 31.70 (27.90, 42.10) | 35.50 (28.60, 53.20) | Z=-5.620 | <0.001 |
| BUN, mg/dL, M (Q_1_, Q_3_) | 21.00 (15.00, 34.00) | 17.00 (13.00, 26.00) | 26.00 (17.00, 44.00) | Z=-13.239 | <0.001 |
| Lactate, mmol/L, M (Q_1_, Q_3_) | 1.90 (1.41, 2.60) | 1.79 (1.40, 2.40) | 2.00 (1.50, 2.90) | Z=-6.706 | <0.001 |
| Bicarbonate, mEq/L, Mean ± SD | 22.06 ± 4.03 | 22.71 ± 3.39 | 21.42 ± 4.49 | t=6.92 | <0.001 |
| Sodium, mEq/L, Mean ± SD | 136.60 ± 4.83 | 136.23 ± 4.11 | 136.97 ± 5.44 | t=-3.27 | 0.001 |
| Potassium, mEq/L, Mean ± SD | 4.42 ± 0.78 | 4.43 ± 0.75 | 4.41 ± 0.81 | t=0.43 | 0.667 |
| Chloride, mEq/L, Mean ± SD | 103.80 ± 6.23 | 104.51 ± 5.77 | 103.09 ± 6.59 | t=4.89 | <0.001 |
| Time stayed in ICU, days, M (Q_1_, Q_3_) | 2.73 (1.54, 4.96) | 2.19 (1.35, 3.76) | 3.19 (1.90, 6.19) | Z=-9.881 | <0.001 |
| Follow-up time, days, M (Q_1_, Q_3_) | 6.24 (4.33, 10.81) | 5.74 (4.29, 8.68) | 7.41 (4.63, 12.23) | Z=-6.258 | <0.001 |
| Outcome, n (%) |  |  |  | χ^2^=86.285 | <0.001 |
| Survival | 1557 (86.26) | 846 (93.79) | 711 (78.74) |  |  |
| Mortality | 248 (13.74) | 56 (6.21) | 192 (21.26) |  |  |
| Glucose, mg/dL, M (Q_1_, Q_3_) | 142.00 (117.00, 188.00) | 127.00 (108.00, 152.00) | 171.00 (131.00, 222.00) | Z=-17.182 | <0.001 |
| Lymphocytes, K/uL, M (Q_1_, Q_3_) | 1.38 (0.85, 2.06) | 2.03 (1.58, 2.71) | 0.85 (0.58, 1.17) | Z=31.775 | <0.001 |
| GLR level, M (Q_1_, Q_3_) | 106.14 (66.30, 198.41) | 66.17 (48.97, 84.22) | 198.41 (138.84, 312.50) | Z=-36.783 | <0.001 |

AMI: acute myocardial infarction, GLR: glucose-to-lymphocyte ratio, SD: standard deviation, M: median, Q_1_:1st Quartile, Q_3_:3st Quartile, MBP: mean blood pressure, bpm: beats per minute, SAPS-II: Simplified Acute Physiology Score II, SOFA: Sequential Organ Failure Assessment, GCS: Glasgow Coma Scale score, WBC: white blood cell, RDW: red cell distribution width, INR: international normalized ratio, PT: prothrombin time, PTT: partial thromboplastin time, BUN: blood urea nitrogen

t: t-test, Z: rank sum test, χ2: chi-square test, -: Fisher exact test

**Screening of the potential confounding factors**

Table 2 was the univariate analysis to explore the potential confounding factors of in-hospital mortality in AMI patients. The results showed that age [HR=1.02, 95% CI: (1.01-1.03)], vasopressor use [HR=2.81, 95% CI: (1.98-3.99)], receiving renal replacement therapy [HR=1.54, 95% CI: (1.14-2.09)], cardiogenic shock [HR=1.96, 95% CI: (1.50-2.57)], weight [HR=0.99, 95% CI: (0.99-0.99)], respiratory rate [HR=1.06, 95% CI: (1.04-1.08)], heart rate [HR=1.01, 95% CI: (1.01-1.02)], SPO_2_ [HR=0.93, 95% CI: (0.90-0.96)], SAPS-II scores [HR=1.04, 95% CI: (1.03-1.05)], SOFA score [HR=1.16, 95% CI: (1.13-1.19)], CCI [HR=1.10, 95% CI: (1.05-1.16)], neutrophil [HR=1.01, 95% CI: (1.01-1.02)], RDW [HR=1.11, 95% CI: (1.06-1.16)], creatinine [HR=1.12, 95% CI: (1.05-1.19)], INR [HR=1.09, 95% CI: (1.01-1.18)], PT [HR=1.01, 95% CI: (1.01-1.02)], PTT [HR=1.01, 95% CI: (1.01-1.01)], BUN [HR=1.01, 95% CI: (1.01-1.02)], lactate [HR=1.13, 95% CI: (1.09-1.17)], bicarbonate [HR=0.93, 95% CI: (0.90-0.95)], sodium [HR=1.04, 95% CI: (1.01-1.06)], chloride [HR=0.98, 95% CI: (0.96-0.99)], received coronary artery bypass graft [HR=0.28, 95% CI: (0.15-0.53)] and β blockers usage [HR=0.54, 95% CI: (0.41-0.73)] were related to in-hospital mortality in AMI patients.

**Table 2. Univariate analysis to screen the potential confounding factors of in-hospital mortality in AMI patients**

| Variables | HR (95% CI) | *P* |
| --- | --- | --- |
| Age | 1.02 (1.01-1.03) | <0.001 |
| Gender |  |  |
| Female | Ref |  |
| Male | 0.92 (0.71-1.19) | 0.511 |
| Race |  |  |
| Black | Ref |  |
| White | 0.87 (0.53-1.42) | 0.581 |
| Others | 0.85 (0.47-1.57) | 0.611 |
| Unknown | 1.15 (0.67-1.97) | 0.603 |
| Insurance |  |  |
| Medicaid | Ref |  |
| Medicare | 1.52 (0.77-2.99) | 0.223 |
| Others | 1.26 (0.64-2.50) | 0.504 |
| Ventilation |  |  |
| No | Ref |  |
| Yes | 2.40 (0.99-5.83) | 0.052 |
| Vasopressor |  |  |
| No | Ref |  |
| Yes | 2.81 (1.98-3.99) | <0.001 |
| Renal replacement therapy |  |  |
| No | Ref |  |
| Yes | 1.54 (1.14-2.09) | 0.005 |
| Atrial fibrillation |  |  |
| No | Ref |  |
| Yes | 1.18 (0.90-1.53) | 0.226 |
| Cardiogenic shock |  |  |
| No | Ref |  |
| Yes | 1.96 (1.50-2.57) | <0.001 |
| Hypertension |  |  |
| No | Ref |  |
| Yes | 0.82 (0.64-1.05) | 0.120 |
| Weight | 0.99 (0.99-0.99) | 0.045 |
| MBP | 1.00 (0.99-1.01) | 0.859 |
| Respiratory rate | 1.06 (1.04-1.08) | <0.001 |
| Heart rate | 1.01 (1.01-1.02) | <0.001 |
| Temperature | 0.94 (0.78-1.14) | 0.533 |
| SPO_2_ | 0.93 (0.90-0.96) | <0.001 |
| SAPS-II scores | 1.04 (1.03-1.05) | <0.001 |
| SOFA score | 1.16 (1.13-1.19) | <0.001 |
| Charlson comorbidity index | 1.10 (1.05-1.16) | <0.001 |
| Neutrophil | 1.01 (1.01-1.02) | 0.019 |
| Platelet | 1.00 (1.00-1.00) | 0.280 |
| Haemoglobin | 0.99 (0.94-1.04) | 0.729 |
| RDW | 1.11 (1.06-1.16) | <0.001 |
| Creatinine | 1.12 (1.05-1.19) | <0.001 |
| INR | 1.09 (1.01-1.18) | 0.040 |
| PT | 1.01 (1.01-1.02) | 0.020 |
| PTT | 1.01 (1.01-1.01) | <0.001 |
| BUN | 1.01 (1.01-1.02) | <0.001 |
| Lactate | 1.13 (1.09-1.17) | <0.001 |
| Bicarbonate | 0.93 (0.90-0.95) | <0.001 |
| Sodium | 1.04 (1.01-1.06) | 0.002 |
| Potassium | 0.94 (0.81-1.10) | 0.462 |
| Chloride | 0.98 (0.96-0.99) | 0.013 |
| Percutaneous coronary intervention |  |  |
| No | Ref |  |
| Yes | 0.93 (0.58-1.49) | 0.769 |
| Coronary artery bypass graft |  |  |
| No | Ref |  |
| Yes | 0.28 (0.15-0.53) | <0.001 |
| Beta-blockers |  |  |
| No | Ref |  |
| Yes | 0.54 (0.41-0.73) | <0.001 |

AMI: acute myocardial infarction, HR: hazard ratio, CI: confidence interval, Ref: reference, MBP: mean blood pressure, SAPS-II: Simplified Acute Physiology Score II, SOFA: Sequential Organ Failure Assessment, RDW: red cell distribution width, INR: international normalized ratio, PT: prothrombin time, PTT: partial thromboplastin time, BUN: blood urea nitrogen

**Association between GLR levels and in-hospital mortality in AMI patients**

We further explored the relationship between GLR and in-hospital mortality in patients with AMI (Table 3). Multivariate model adjusted for the variables selected by step-to-step regression based on those that have significant difference in the univariate analysis (Table 2), including vasopressor, renal replacement therapy, weight, respiratory rate, SPO_2_, SAPS-II scores, RDW, PTT, BUN, lactate, coronary artery bypass graft and β blockers. Compared to the low level of GLR, a high GLR level was linked to an increased risk of in-hospital mortality in AMI patients [HR=1.70, 95% CI: (1.24-2.34)]. Figure 2 was the RCS curve of GLR and in-hospital mortality risk demonstrating that with the increase of GLR, HR value also increases simultaneously. When GLR < 104.6643, the curve was always below HR=1, while GLR >104.6643 crossed HR=1 and raised differently above HR=1. Nonlinear Test results indicated that the RCS curve is nonlinear (*P*<0.0001). We further plotted the KM curve of GLR levels and the probability of survival in AMI patients (Figure 3). From the KM curve, the in-hospital survival rate of the high GLR level group was lower than that of the low GLR level group (*P*<0.0001), representing a significant difference in survival probability between the two groups.

**Table 3. Association between GLR level and in-hospital mortality in AMI patients**

| Variables | Low GLR level  HR (95% CI) | *P* | High GLR level  HR (95% CI) | *P* |
| --- | --- | --- | --- | --- |
| Univariate model | Ref | - | 2.64 (1.95-3.56) | <0.001 |
| Multivariate model | Ref | - | 1.70 (1.24-2.34) | 0.001 |

GLR: glucose-to-lymphocyte ratio, AMI: acute myocardial infarction, HR: hazard ratio, CI: confidence interval, Ref: reference

Multivariate model adjusted for vasopressor, renal replacement therapy, weight, respiratory rate, SPO_2_, SAPS-II scores, RDW, PTT, BUN, lactate, coronary artery bypass graft and β blockers.

**Association between GLR levels and in-hospital mortality in vasopressor use, SOFA scores, renal replacement therapy, gender, coronary artery bypass graft, β blockers and age subgroups**

We also analysed the relationship between GLR and in-hospital mortality in different subgroups (Figure 4). The results revealed that high GLR level was associated with an increased risk of in-hospital mortality, especially in male AMI patients without renal replacement therapy, coronary artery bypass graft, without β blockers use and those aged ≥ 65 years old (HR>1). Moreover, in AMI patients with SOFA score <6 and ≥ six and used vasopressor use or not, high GLR level was both related to the increased risk of in-hospital mortality (HR>1).

**Comparison of the effect of GLR, glucose and lymphocytes on predicting in-hospital mortality**

To further explore the predictive value of GLR, glucose and lymphocytes on in-hospital mortality in AMI patients, we observed the change in AUC of GLR, glucose and lymphocytes 14 days after AMI patients were admitted to the ICU (Figure 5). The AUC of GLR was consistently higher than that of glucose and lymphocytes, and the graphical trend was basically in equilibrium, which indicates that the predictive value of GLR was superior to using glucose or lymphocytes alone.

**Discussion**

In this study, we analyzed the association between GLR and in-hospital mortality and assessed the predicting value of GLR in the prognosis of AMI patients. The results showed that compared to the low level of GLR, high GLR level was linked to the increased risk of in-hospital mortality in AMI patients, especially in patients without renal replacement therapy, male, without coronary artery bypass graft, without β blockers use, aged ≥ 65 years old, SOFA score <6 and ≥ 6 and used vasopressor use or not. Moreover, the predictive value in in-hospital mortality of GLR was superior to glucose or lymphocytes alone.

To our knowledge, no previous studies have reported the association between GLR and the risk of in-hospital mortality in AMI patients. GLR is a recent biomarker that combined the inflammatory indicator lymphocyte and blood glucose level to predict prognosis in some diseases. Zhang et al. [14] indicated that GLR is an independent prognostic factor for pancreatic ductal adenocarcinoma patients undergoing curative resection. Zhong et al. [18] found that GLR is an independent prognostic factor for patients with pancreatic cancer. Another study demonstrated that GLR before sorafenib treatment is a new prognostic biomarker that may predict survival in advanced hepatocellular carcinoma [15]. As far as we know, the present study demonstrated that GLR is a potential prognostic marker to predict outcomes in critically ill patients with AMI according to the analysis multivariate Cox proportional hazard model analysis results. We then compared the predictive abilities of GLR with single glucose and lymphocytes by using the AUC value. The results indicated that the AUC of GLR was higher than the other two indexes. Moreover, the survival KM curve of GLR depicted good performance for predicting in-hospital mortality. Hence, the present study verified a potential good prognosis factor of GLR in predicting in-hospital mortality in AMI patients.

The exact mechanism to explain the link between a high levels of GLR and in-hospital mortality in patients with AMI is largely unknown. Hyperglycemia was reported to be positively correlated with higher mortality in AMI patients, independent of diabetes status [12]. In the early stage of AMI, hyperglycemia promotes prethrombotic rate, increases inflammation and sympathetic nervous system activity, worsens endothelial function, induces oxidative stress to release reactive oxygen species, and thus exacerbates coronary artery damage [10, 11]. Accumulating studies have reported that hyperglycemia on admission was positively correlated with poor prognosis during hospitalization [19, 20]. On the other hand, as indicators of immunity, decreased lymphocyte counts indicate immunity injury [21]. It has been reported that lymphocyte count plays a crucial role in regulating inflammatory responses at all stages of atherosclerosis [8] and is associated with AMI occurrence and prognosis [9]. Migration of activated lymphocytes to the inflammatory site in vivo, such as the pancreas and lungs,deplete circulating lymphocytes [22]. Hence, it is of great value to consider that GLR may reflect the depressed immunity and synergistic effect of hyperglycemia in patients with AMI.

In the present study, high GLR levels showed a significant association with increased risk of in-hospital mortality in male AMI patients, aged ≥ 65 years old and without conventional treatment. Both glucose tolerance and adaptive immune function exhibit significant age-related alterations. A study in old mice showed that T lymphocyte depletion ameliorates age-related metabolic impairments [23]. In our study, the high risk of elderly AMI patients might be attributed to their poor overall health and of increased complications. Therefore, the elderly are becoming an increasingly important subpopulation that requires special attention in light of health and social issues. According to the previous study, AMI is more common in men, and male is an independent cardiovascular risk factor [24]. However, women and the elderly are significantly underrepresented in clinical trials due to the higher incidence of cardiovascular disease in men and recruitment problems among older people and women [25, 26]. Females in animal models are also much less readily used because of the potential impact of variation in sex hormone levels during the hormonal cycle [27]. Moreover, AMI patients without conventional treatment may have a higher risk of in-hospital mortality. In a study by Marenzi et al. [28], renal replacement therapy use was associated with a significant increase in in-hospital mortality and a more complicated clinical course in AMI patients. Malmberg et al. [29] found in-hospital mortality to be significantly higher when coronary artery bypass graft was performed for patients with AMI compared to patients with stable coronary artery disease. Another study indicated that β blockers might not be related to all-cause mortality in AMI patients [30]. In this study, we found that the assessment of GLR in patients not receiving conventional AMI treatment is of ,more significant concern. However, the precise mechanisms of the effect of treatment of AMI patients on the GLR and in-hospital mortality are unclear.

This study is the first to investigate the association between GLR level after admission to ICU and the risk of in-hospital mortality in patients with AMI and to report its short-term predictive value. GLR has the advantages of being fast, cheap and easy to obtain, which has specific reference values for rapid identification of high-risk patients in the clinic. There are some limitations of this study. As a single-centre retrospective study, this study inevitably has a particular selection bias. The MIMIC-IV database limited the data we used, and confounding factors such as myocardial infarction size that may affect prognosis were challenging to consider. In addition, the number of mortalities in this study is relatively small, so further prospective studies with large samples are needed.

**Conclusion**

GLR may be a potential prognostic predictor of AMI that can help identify and identify and manage the high-risk populations in clinicidentify and manage the high-risk populations in clinics.

**Acknowledgment**

None.

**Reference**

1.	Statescu C, Anghel L, Tudurachi BS, Leonte A, Benchea LC, Sascau RA. From Classic to Modern Prognostic Biomarkers in Patients with Acute Myocardial Infarction. Int J Mol Sci. 2022;23(16). Epub 2022/08/27. doi: 10.3390/ijms23169168. PubMed PMID: 36012430; PubMed Central PMCID: PMCPMC9409468.

2.	Radziejewska J, Fraczkowski M, Slawuta A, Panaszek B. Can the in-hospital mortality rate in patients with ST-elevation myocardial infarctions be lowered any further? Adv Clin Exp Med. 2021;30(1):23-7. Epub 2020/12/31. doi: 10.17219/acem/128746. PubMed PMID: 33377318.

3.	Li J, Li X, Wang Q, Hu S, Wang Y, Masoudi FA, et al. ST-segment elevation myocardial infarction in China from 2001 to 2011 (the China PEACE-Retrospective Acute Myocardial Infarction Study): a retrospective analysis of hospital data. Lancet. 2015;385(9966):441-51. Epub 2014/06/28. doi: 10.1016/S0140-6736(14)60921-1. PubMed PMID: 24969506; PubMed Central PMCID: PMCPMC4415374.

4.	Murugiah K, Wang Y, Nuti SV, Li X, Li J, Zheng X, et al. Are non-ST-segment elevation myocardial infarctions missing in China? Eur Heart J Qual Care Clin Outcomes. 2017;3(4):319-27. Epub 2017/09/28. doi: 10.1093/ehjqcco/qcx025. PubMed PMID: 28950309.

5.	Trinh K, Julovi SM, Rogers NM. The Role of Matrix Proteins in Cardiac Pathology. Int J Mol Sci. 2022;23(3). Epub 2022/02/16. doi: 10.3390/ijms23031338. PubMed PMID: 35163259; PubMed Central PMCID: PMCPMC8836004.

6.	Wang Y, Yuan M, Ma Y, Shao C, Wang Y, Qi M, et al. The Admission (Neutrophil+Monocyte)/Lymphocyte Ratio Is an Independent Predictor for In-Hospital Mortality in Patients With Acute Myocardial Infarction. Front Cardiovasc Med. 2022;9:870176. Epub 2022/04/26. doi: 10.3389/fcvm.2022.870176. PubMed PMID: 35463771; PubMed Central PMCID: PMCPMC9021423.

7.	Soehnlein O, Libby P. Targeting inflammation in atherosclerosis - from experimental insights to the clinic. Nat Rev Drug Discov. 2021;20(8):589-610. Epub 2021/05/13. doi: 10.1038/s41573-021-00198-1. PubMed PMID: 33976384; PubMed Central PMCID: PMCPMC8112476 disrupting CCL5-HNP1 heteromers, and also receives funding from Novo Nordisk to study chronopharmacological treatment strategies in cardiovascular disease. P.L. is a member of the scientific advisory board for Amgen, Corvidia Therapeutics, DalCor Pharmaceuticals, Kowa Pharmaceuticals, Olatec Therapeutics, Medimmune, Novartis and XBiotech, Inc. P.L.'s laboratory has received research funding in the past 2 years from Novartis. P.L. is on the Board of Directors of XBiotech, Inc. P.L. has a financial interest in Xbiotech, a company developing therapeutic human antibodies. P.L.'s interests were reviewed and are managed by Brigham and Women's Hospital and Partners HealthCare in accordance with their conflict of interest policies.

8.	Ait-Oufella H, Salomon BL, Potteaux S, Robertson AK, Gourdy P, Zoll J, et al. Natural regulatory T cells control the development of atherosclerosis in mice. Nat Med. 2006;12(2):178-80. Epub 2006/02/08. doi: 10.1038/nm1343. PubMed PMID: 16462800.

9.	Shiyovich A, Gilutz H, Plakht Y. White Blood Cell Subtypes Are Associated with a Greater Long-Term Risk of Death after Acute Myocardial Infarction. Tex Heart Inst J. 2017;44(3):176-88. Epub 2017/08/02. doi: 10.14503/THIJ-16-5768. PubMed PMID: 28761398; PubMed Central PMCID: PMCPMC5505396.

10.	D'Onofrio N, Sardu C, Paolisso P, Minicucci F, Gragnano F, Ferraraccio F, et al. MicroRNA-33 and SIRT1 influence the coronary thrombus burden in hyperglycemic STEMI patients. J Cell Physiol. 2020;235(2):1438-52. Epub 2019/07/12. doi: 10.1002/jcp.29064. PubMed PMID: 31294459.

11.	Kitano D, Takayama T, Nagashima K, Akabane M, Okubo K, Hiro T, et al. A comparative study of time-specific oxidative stress after acute myocardial infarction in patients with and without diabetes mellitus. BMC Cardiovasc Disord. 2016;16:102. Epub 2016/05/25. doi: 10.1186/s12872-016-0259-6. PubMed PMID: 27216220; PubMed Central PMCID: PMCPMC4877735.

12.	Paolisso P, Foa A, Bergamaschi L, Angeli F, Fabrizio M, Donati F, et al. Impact of admission hyperglycemia on short and long-term prognosis in acute myocardial infarction: MINOCA versus MIOCA. Cardiovasc Diabetol. 2021;20(1):192. Epub 2021/09/26. doi: 10.1186/s12933-021-01384-6. PubMed PMID: 34560876; PubMed Central PMCID: PMCPMC8464114.

13.	Zhang Y, Zhang S. Prognostic value of glucose-to-lymphocyte ratio in critically ill patients with acute respiratory distress syndrome: A retrospective cohort study. J Clin Lab Anal. 2022;36(5):e24397. Epub 2022/04/01. doi: 10.1002/jcla.24397. PubMed PMID: 35358348; PubMed Central PMCID: PMCPMC9102764.

14.	Zhang Y, Xu Y, Wang D, Kuang T, Wu W, Xu X, et al. Prognostic value of preoperative glucose to lymphocyte ratio in patients with resected pancreatic cancer. Int J Clin Oncol. 2021;26(1):135-44. Epub 2020/09/23. doi: 10.1007/s10147-020-01782-y. PubMed PMID: 32959232.

15.	Yilmaz A, Simsek M, Hannarici Z, Buyukbayram ME, Bilici M, Tekin SB. The importance of the glucose-to-lymphocyte ratio in patients with hepatocellular carcinoma treated with sorafenib. Future Oncol. 2021;17(33):4545-59. Epub 2021/08/26. doi: 10.2217/fon-2021-0457. PubMed PMID: 34431372.

16.	Jhou HJ, Chen PH, Yang LY, Chang SH, Lee CH. Plasma Anion Gap and Risk of In-Hospital Mortality in Patients with Acute Ischemic Stroke: Analysis from the MIMIC-IV Database. J Pers Med. 2021;11(10). Epub 2021/10/24. doi: 10.3390/jpm11101004. PubMed PMID: 34683145; PubMed Central PMCID: PMCPMC8541378.

17.	Johnson AE, Stone DJ, Celi LA, Pollard TJ. The MIMIC Code Repository: enabling reproducibility in critical care research. J Am Med Inform Assoc. 2018;25(1):32-9. Epub 2017/10/17. doi: 10.1093/jamia/ocx084. PubMed PMID: 29036464; PubMed Central PMCID: PMCPMC6381763.

18.	Zhong A, Cheng CS, Kai J, Lu R, Guo L. Clinical Significance of Glucose to Lymphocyte Ratio (GLR) as a Prognostic Marker for Patients With Pancreatic Cancer. Front Oncol. 2020;10:520330. Epub 2020/10/30. doi: 10.3389/fonc.2020.520330. PubMed PMID: 33117673; PubMed Central PMCID: PMCPMC7561421.

19.	Whitcomb BW, Pradhan EK, Pittas AG, Roghmann MC, Perencevich EN. Impact of admission hyperglycemia on hospital mortality in various intensive care unit populations. Crit Care Med. 2005;33(12):2772-7. Epub 2005/12/15. doi: 10.1097/01.ccm.0000189741.44071.25. PubMed PMID: 16352959.

20.	Egi M, Bellomo R, Stachowski E, French CJ, Hart GK, Hegarty C, et al. Blood glucose concentration and outcome of critical illness: the impact of diabetes. Crit Care Med. 2008;36(8):2249-55. Epub 2008/07/31. doi: 10.1097/CCM.0b013e318181039a. PubMed PMID: 18664780.

21.	Chen ZQ, Yu XS, Mao LJ, Zheng R, Xue LL, Shu J, et al. Prognostic value of neutrophil-lymphocyte ratio in critically ill patients with cancer: a propensity score matching study. Clin Transl Oncol. 2021;23(1):139-47. Epub 2020/05/31. doi: 10.1007/s12094-020-02405-8. PubMed PMID: 32472452.

22.	Bhatia M, Wong FL, Cao Y, Lau HY, Huang J, Puneet P, et al. Pathophysiology of acute pancreatitis. Pancreatology. 2005;5(2-3):132-44. Epub 2005/04/26. doi: 10.1159/000085265. PubMed PMID: 15849484.

23.	Trott DW, Islam MT, Buckley DJ, Donato AJ, Dutson T, Sorensen ES, et al. T lymphocyte depletion ameliorates age-related metabolic impairments in mice. Geroscience. 2021;43(3):1331-47. Epub 2021/04/25. doi: 10.1007/s11357-021-00368-4. PubMed PMID: 33893902; PubMed Central PMCID: PMCPMC8190228.

24.	Arora S, Stouffer GA, Kucharska-Newton AM, Qamar A, Vaduganathan M, Pandey A, et al. Twenty Year Trends and Sex Differences in Young Adults Hospitalized With Acute Myocardial Infarction. Circulation. 2019;139(8):1047-56. Epub 2018/12/28. doi: 10.1161/CIRCULATIONAHA.118.037137. PubMed PMID: 30586725; PubMed Central PMCID: PMCPMC6380926.

25.	Amuthan R, Curtis AB. Sex-Specific Considerations in Drug and Device Therapy of Cardiac Arrhythmias: JACC Focus Seminar 6/7. J Am Coll Cardiol. 2022;79(15):1519-29. Epub 2022/04/16. doi: 10.1016/j.jacc.2021.11.066. PubMed PMID: 35422248.

26.	Zeitler EP, Poole JE, Albert CM, Al-Khatib SM, Ali-Ahmed F, Birgersdotter-Green U, et al. Arrhythmias in Female Patients: Incidence, Presentation and Management. Circ Res. 2022;130(4):474-95. Epub 2022/02/18. doi: 10.1161/CIRCRESAHA.121.319893. PubMed PMID: 35175839.

27.	Okninska M, Maczewski M, Mackiewicz U. Ventricular arrhythmias in acute myocardial ischaemia-Focus on the ageing and sex. Ageing Res Rev. 2022;81:101722. Epub 2022/08/30. doi: 10.1016/j.arr.2022.101722. PubMed PMID: 36038114.

28.	Marenzi G, Cosentino N, Marinetti A, Leone AM, Milazzo V, Rubino M, et al. Renal replacement therapy in patients with acute myocardial infarction: Rate of use, clinical predictors and relationship with in-hospital mortality. Int J Cardiol. 2017;230:255-61. Epub 2017/01/04. doi: 10.1016/j.ijcard.2016.12.130. PubMed PMID: 28043673.

29.	Malmberg M, Gunn J, Rautava P, Sipila J, Kyto V. Outcome of acute myocardial infarction versus stable coronary artery disease patients treated with coronary bypass surgery. Ann Med. 2021;53(1):70-7. Epub 2020/09/03. doi: 10.1080/07853890.2020.1818118. PubMed PMID: 32875916; PubMed Central PMCID: PMCPMC7877950.

30.	Wen XS, Luo R, Liu J, Duan Q, Qin S, Xiao J, et al. Short-term/long-term prognosis with or without beta-blockers in patients without heart failure and with preserved ejection fraction after acute myocardial infarction: a multicenter retrospective cohort study. BMC Cardiovasc Disord. 2022;22(1):193. Epub 2022/04/28. doi: 10.1186/s12872-022-02631-8. PubMed PMID: 35473676; PubMed Central PMCID: PMCPMC9044853.

**Figure legends**

Figure 1. Flowchart of the data screening.

Figure 2. The RCS curve of GLR and in-hospital mortality risk.

Figure 3. The KM curve of GLR levels and probability of survival in AMI patients.

Figure 4. The relationship between GLR and in-hospital mortality in subgroups of vasopressor use, SOFA scores, renal replacement therapy, gender, coronary artery bypass graft, β blockers and age.

Figure 5. The predictive value of GLR, glucose and lymphocytes on in-hospital mortality in 14 days after AMI patients admitted in the ICU.
