# Supplementary material for: Association between glucose-to-lymphocyte ratio and in-hospital mortality in acute myocardial infarction patients
Source: PLoS One. 2023 Dec 7;18(12):e0295602. doi: 10.1371/journal.pone.0295602 (PMC10703328; doi:10.1371/journal.pone.0295602)
Supplement: Attachment — Submitted filename: Response to reviewers comments.docx [file pone.0295602.s003.docx]

**Responses to Reviewers**

Dear Editor and Reviewers,

Thank you very much for your time to review and valuable comments on our manuscript. We have carefully studied all the comments and suggestions, and then revised our paper accordingly. The point-by-point responses to the comments are as follow. We hope our responses adequately address the comments and the revised manuscript can achieve your standards. Thank you for your consideration.

**Responses to Reviewer #1’s comments**

The manuscript has potential but it is flawed with a lot of typographical error and needs a lot of editing.

Response: Thank you for your approval and valuable comments. We have checked and revised the typographical error in the manuscript, and invited a native English speaker to help us revise the article.

Line 8: Check the spelling of the name of the university

Response: We have revised the name of the university.

All other comments have been made in the attached document

Response: We have accepted all the amendments and revised other mistakes to make the manuscript clearer.

**Responses to Reviewer #2’s comments**

The paper is well-prepared, displaying a solid grounding in its subject matter. The authors have engaged thoroughly with existing literature, providing a clear context for their study. This offers readers a comprehensible background and helps to position the research within the broader academic conversation. The methodology section of the paper is clear and straightforward, showing a systematic approach to the research questions. It appears that careful planning has been applied to ensure that the research process is both robust and reproducible.

The statistical analysis presented in the study is transparent and accessible. The findings seem to be the result of a meticulous analysis process, making them both reliable and valuable for the academic community. This adds a layer of credibility to the paper and enhances its contribution to the field. The conclusion drawn by the authors is engaging. It manages to capture the essence of the research findings and presents them in a way that is both interesting and informative. It provides a solid ending to the paper, leaving the reader with a clear understanding of the significance of the research.

In considering the overall presentation and content of the paper, it appears to be a well-rounded piece of academic work. The simplicity of language and clarity of expression make the paper accessible to a broad audience, ensuring that it can be easily understood and appreciated by both experts in the field and those with a more general interest. Given these considerations, it seems that the paper is a worthy candidate for publication, as it can make a meaningful contribution to ongoing scholarly discussions and investigations within the field.

Response: Thank you very much for your approval and valuable comments. We have revised the grammatical mistake in the manuscript to make it easier to read and understand.
